# The Batten Disease *Palmitoyl Protein Thioesterase 1* Gene Regulates Neural Specification and Axon Connectivity during *Drosophila* Embryonic Development

**DOI:** 10.1371/journal.pone.0014402

**Published:** 2010-12-22

**Authors:** Quynh Chu-LaGraff, Cassandra Blanchette, Patrick O'Hern, Cassandra Denefrio

**Affiliations:** Department of Biology, Union College, Schenectady, New York, United States of America; Yale School of Medicine, United States of America

## Abstract

Palmitoyl Protein Thioesterase 1 (PPT1) is an essential lysosomal protein in the mammalian nervous system whereby defects result in a fatal pediatric disease called Infantile Neuronal Ceroids Lipofuscinosis (INCL). Flies bearing mutations in the *Drosophila* ortholog *Ppt1* exhibit phenotypes similar to the human disease: accumulation of autofluorescence deposits and shortened adult lifespan. Since INCL patients die as young children, early developmental neural defects due to the loss of PPT1 are postulated but have yet to be elucidated. Here we show that *Drosophila Ppt1* is required during embryonic neural development. *Ppt1* embryos display numerous neural defects ranging from abnormal cell fate specification in a number of identified precursor lineages in the CNS, missing and disorganized neurons, faulty motoneuronal axon trajectory, and discontinuous, misaligned, and incorrect midline crossings of the longitudinal axon bundles of the ventral nerve cord. Defects in the PNS include a decreased number of sensory neurons, disorganized chordotonal neural clusters, and abnormally shaped neurons with aberrant dendritic projections. These results indicate that *Ppt1* is essential for proper neuronal cell fates and organization; and to establish the local environment for proper axon guidance and fasciculation. Ppt1 function is well conserved from humans to flies; thus the INCL pathologies may be due, in part, to the accumulation of various embryonic neural defects similar to that of *Drosophila*. These findings may be relevant for understanding the developmental origin of neural deficiencies in INCL.

## Introduction

Infantile Neuronal Ceroids Lipofuscinoses (INCL) belongs to a group of lysosomal storage disorders characterized by the fatal progressive deterioration of the visual and central nervous system, and the accumulation of abnormal autofluorescent storage materials in the brain [1], [2]. INCL, the most severe form, results from defects in the protein Palmitoyl Protein Thioesterase 1 (PPT1) which encodes a lysosomal thioesterase that cleaves long fatty acids-most likely palmitate-attached to the cysteine residues of S-acylated protein substrates [3], [4]. In mammals, PPT1 expression is found in all cell types at varying quantities with the highest levels in the brain, eye, and spleen [5], [6]. In addition to being found in lysosomal compartments of neurons, the protein is also expressed in the pre-synaptic compartments [7], [8], [9].

Although ubiquitously expressed, PPT1 deficiency affects only the development and maintenance of cortical neurons in the nervous system. Afflicted children are normal at birth; but exhibit progressive cognitive and motor deficits, seizures, and ocular deterioration and eventual blindness by the age of 3; after which INCL children remain in a vegetative state until death in their teens. Microarray studies reveal that changes in gene expression can be detected at 10 week-old post-natal PPT1 knock-out mice brains, a time prior to neurodegenerative symptoms [10]. These findings suggest that the loss of this protein may have embryonic developmental consequences prior to being symptomatic. If PPT1 does have a role during embryonic neurogenesis, then the elucidation of the fundamental cellular pathways requiring PPT1 during development will be critical.

Using *Drosophila*, we investigate the role of PPT1 during the development of the embryonic nervous system. Previous studies indicated that the removal of the *Drosophila* ortholog *Ppt1* results in phenotypes similar to human INCL [11], [12]. *Ppt1* loss-of-function (LOF) flies lacking Ppt1 enzymatic activity display some aspects of INCL disease: reduced life expectancy and an age-dependent accumulation of autofluorescent storage material in the adult CNS. Overexpression of Ppt1 in the larval visual system leads to increased cell death [13]. A dominant gain-of-function modifier screen for genes interacting with *Ppt1* suggests that it has a role in synaptic developmental pathways and the regulation of synaptic vesicle endocytosis [14].

Here, we investigate the potential role of *Ppt1* during the generation of the nervous system by focusing on discreet identified neuronal cell lineages. We hypothesize that while *Ppt1* mutants may not show detectable adult brain abnormality, early defects at the cellular level may be present in the form of altered cell fate specification, proliferation, and axon guidance and connectivity. Our results support this hypothesis and indicate that the loss of *Ppt1* has consequences much earlier than previously described. *Ppt1* defects lead to mis-specification of identified CNS and PNS neural precursor cell fates, an abnormal complement of neural precursors and neurons, and motoneuronal axonal misrouting and overall defective axon pathfinding and fasciculation. These *Ppt1*-associated embryonic phenotypes may contribute to the shortened lifespan of *Ppt1*-deficient flies. These findings in *Drosophila* may be relevant in identifying the earliest developmental neural defects in INCL afflicted individuals and potential cellular targets for therapeutic interventions.

## Results

### *Drosophila* Ppt1 RNA and protein are expressed at very low/undetectable levels

To ascertain the endogenous RNA Ppt1 expression pattern during development, whole mount *in situ* experiments on various developmentally staged embryos (from 0 hr. to 16 hr), and wing, leg, and eye imaginal discs using digoxygenin-labeled DNA and RNA Ppt1 probes were performed. Both labeled probes reveal low ubiquitous staining indicating that Ppt1 RNA is expressed ubiquitously at a very low level (Figure S1 of Supporting Information). These results support the hypothesis that Ppt1 acts as a metabolic enzyme with housekeeping function with relatively little need for high RNA concentration and/or turnover.

To examine localization of Ppt1 protein, four chicken/rabbit polyclonal antibodies were generated against different regions of Ppt1 that include both internal sequences and the C-terminus. Embryonic, third-instar larvae, and whole adult male and female lysates were used on Western blot to test all crude sera and affinity-purified polyclonal antibodies. Results reveal that the endogenous Ppt1 protein is undetectable under standard conditions (Figure S2). To determine whether the Ppt1 polyclonal sera is specific to Ppt1, Western blot using concentrated S2 Schneider cell lysates, and adult fly head lysates (obtained via a collaboration with Dr. Robert Glaser of Wadsworth Center of NYS Department of Health) isolated from over-expressing UAS:Ppt1;P{GAL4-elav.L} flies were performed. Results indicate that only when Ppt1 is over-expressed at a high level (greater than 10 folds), or is highly enriched in S2 lysates, can a band be detected at the expected size of approximately 28 kD (Figure S2). Since Ppt1 has several conserved glycosylation sites, de-glycosylation experiment using PGNAse F (Invitrogen, Inc.) was carried out to confirm the specificity of the Ppt1 polyclonal sera. Western blot reveals a single band of ∼28kD being shifted to a de-glycosylated band of 26 kD which is similar in size as the mammalian PPT1 (Figure S2; Verkruyse and Hoffmann, 1996). Since Ppt1 protein is only detected when over-expressed on a Western, it indicates that (1) the polyclonal antibodies were successful at detecting endogenous Ppt1 protein; and (2) the protein is expressed at very low levels endogeneously.

To investigate whether the loss of *Ppt1* have consequences during embryonic development, embryos homozygous for three *Ppt1* mutant strains were used: the Ppt1 null allele *Df(1)446-20*, which removes *Ppt1* and three neighboring genes [11]; and two EMS-generated alleles: *Ppt1^A179T^* which contains a point mutation that changed alanine 179 to threonine; and *Ppt1^S77F^* which contains a point mutation that changed serine 77 to phenylalanine [12]. Both EMS alleles exhibit no detectable Ppt1-specific enzyme activity. Additionally, female embryos trans-heterozygous for *Df(1)446-20* and each point mutant alleles were generated in all pair-wise combinations: *Ppt1^A179T^/Df(1)446-20*, *Ppt1^S77F^/Df(1)446-20*, *Ppt1^A179T^/Ppt1^S77F^*. We compared neural development of these embryos to those that are heterozygous for each Ppt1 strains, namely *Df(1)446-20/+*, *Ppt1^A179T^/+*, *and Ppt1^S77F^/+*.

### *Ppt1*-deficient embryos do not display autofluorescence accumulation

INCL human patients and *Ppt1* LOF flies exhibit accumulation of autofluorescent deposits in adult brains due to the loss of protein function. Previous studies indicated that adult Ppt1 mutant fly brains exhibit larger, more abundant autofluorescent deposits than wild type and this accumulation is visible several days after pupation [12]. We also examined 14-day old wild type and Ppt1 LOF S77F adult fly brains for differences in autofluorescent deposits. As in the previous studies, our results showed that there is detectable difference in the levels of autofluorescent deposits. Although wild type adult brains display minimal deposits, Ppt1 LOF brains had more deposits visible at various wavelengths. We asked whether these accumulations are detectable during embryogenesis. Wild type and *Ppt1* LOF stage 8–17 embryos were examined for increased autofluorescent deposits in the developing nervous system. We detected no difference in the level of autofluorescence between wild type and *Ppt1*embryos at all stages examined (data not shown) indicating that the accumulation of abnormal storage materials is age-dependent and that the duration of embryogenesis is not sufficient for build-up to occur.

### *Ppt1* mutants display aberrant cell fate specification in early embryonic CNS development

We addressed the question of whether *Ppt1* is involved in the early events of neurogenesis- namely neural precursor specification and division. We focused on the development of some of the most well characterized neural cell lineages in the fly CNS using the mAb EVE (mAb 2B8). EVE has a defined expression patterns in ganglion mother cells (GMC) and daughter neurons of identified neural precursor lineages NB1-1, 3-3, 4-2, and 7-1 [15], [16], [17], [18]. EVE is expressed in the first-born precursor of NB1-1, GMC1-1a, and its progeny, the aCC and pCC motoneurons; the EL neurons deriving from NB3-3; GMC4-2 and its daughter neurons RP2 motoneuron and its sib from NB4-2, and the U/CQ neurons deriving from NB7-1.

**Figure 1:**
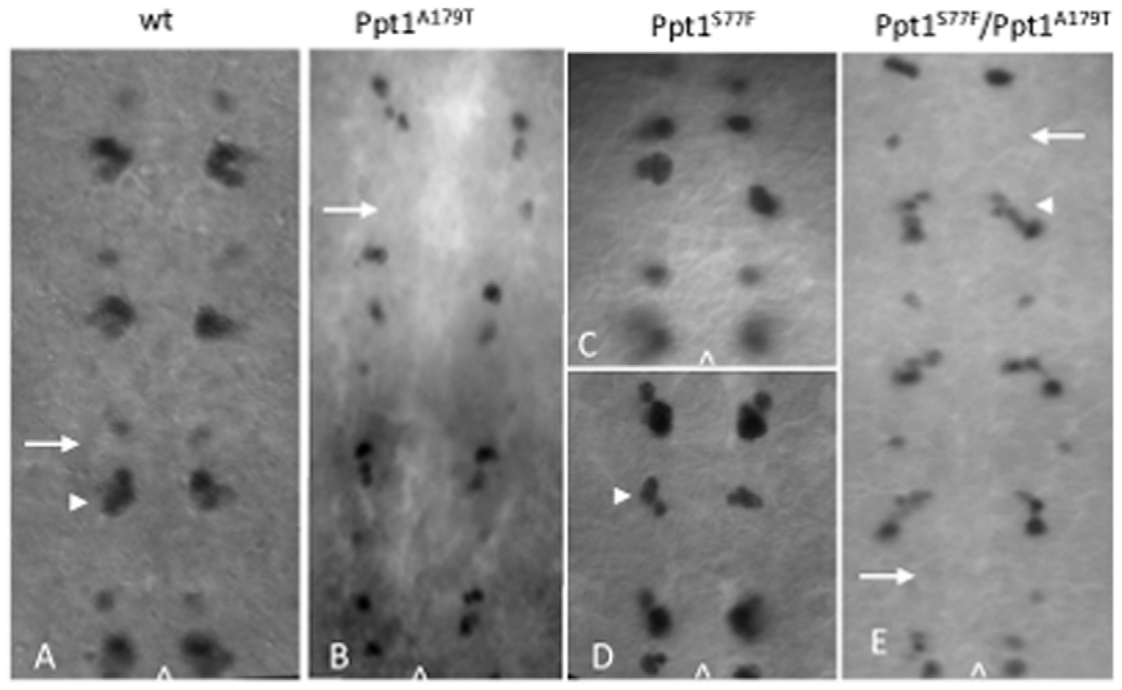
*Ppt1* LOF mutants exhibit abnormal complement of EVE+ neural precursors and neurons. (A) In wild type, EVE is expressed in GMC4-2.a, the neural precursor that give rise to RP2 motoneuron and its sib (arrow); and in a cluster of GMCs that give rise to the aCC, pCC, CQ neurons (arrowhead). (B–E) In *Ppt1* mutants, many hemisegments exhibit a variety of phenotype including the loss of GMC4.2a (arrow in B and E), extra RP2/sib neurons (arrowhead in D), disorganized EVE+ clusters (C), and extra aCC/pCC cells (arrowhead in E). In all panels, embryos are at S11–12 of development; anterior, up; caret, midline.

**Figure 2:**
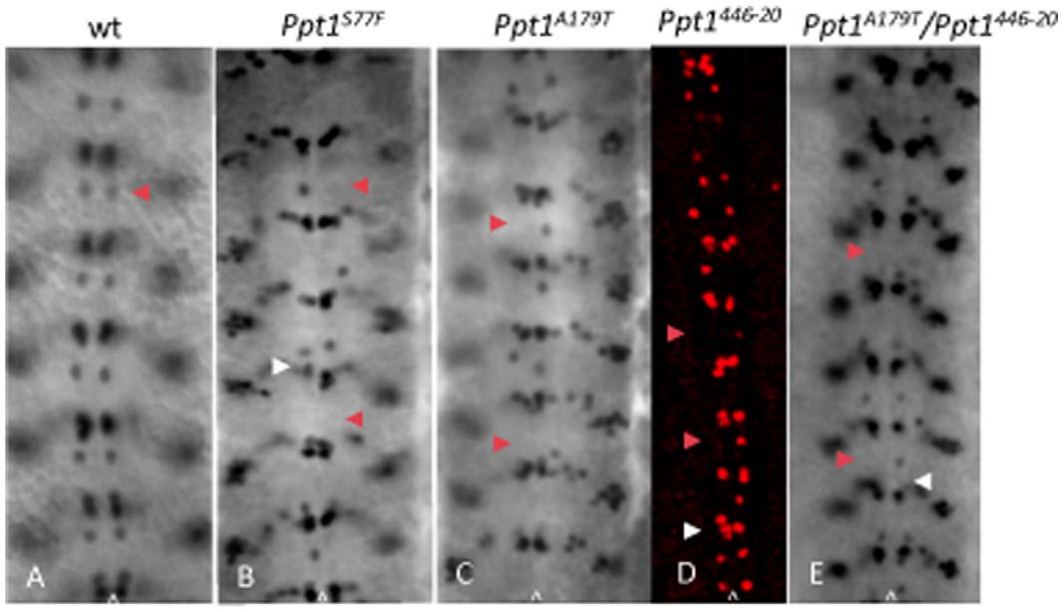
Loss of *Ppt1* results in missing EVE+ RP2 neurons in many hemisegments of S16 embryonic CNS. Arrowhead indicates RP2 neuron in all panels. (A). Wild type EVE+ CNS pattern. (B–E) *Ppt1* LOF mutants exhibit a variety of phenotype including the loss of EVE+ RP2s in some hemisegments (red arrowhead in B–E), disorganized or missing aCC/pCC clusters (white arrowhead in B, D and E). Anterior, up; caret, midline.

Results indicate that *Ppt1* mutant embryos display abnormal complement of EVE+ GMCs and their progeny in many hemisegments, as early as stage 11/12 of embryogenesis. At stage 11–12, EVE is expressed in GMCs that produce the RP2 motoneuron and its sib; aCC/pCC and U/CQ neurons (Figure 1A). *Ppt1* mutant embryos from *Df(1)446-20*, *Ppt1^A179T^*, *and Ppt1^S77F^*, and trans-heterozygous *Ppt1* mutant crosses show a variety of phenotypes. Some embryos display cellular disorganization where EVE+ GMCs are displaced. In others, there is a loss or gain of extra EVE-positive GMCs and neurons in many hemisegments (Figure 1B–E). The most prevalent phenotype is the loss of GMC4-2.a, the parental precursor that will give rise to the RP2 motoneuron. Occasionally, extra U/CQ and aCC/pCC GMCs are also observed as also the presence of extra RP2s or its sib after GMC4-2.a has divided (Figure 1D–E). Overall, 31% (n = 42) of *Ppt1-* embryos display abnormality at stage 12. Later in embryonic development during stage 14–15 when the EVE+ GMCs give rise to EVE+ neurons, the loss of EVE+ RP2s is observed in a small, but significant percentage of hemisegments in *Ppt1*mutants (Figure 2; Table 1). Specifically, over 36% of T1-A8 hemisegments show a loss of RP2s in *Df(1)446-20* embryos. Point mutants and trans-heterozygous embryos show 12–17% loss in all hemisegments (Table 1). The degree of penetrance of this EVE neural phenotype is variable: all *Df(1)446-20* embryos show at least two missing RP2s whereas *Ppt1^A179T^ and Ppt1^S77F^* point mutations range from 32–50%. 63% of embryos from *Ppt1^A179T^×Df(1)446-20* trans-heterozygous crosses and 27% of *Ppt1^S77F^×Df(1)446-20* trans-heterozygous crosses display a partial loss of EVE+ RP2 neurons (Figure 2; Table 2). In contrast, all Ppt1 mutant strains outcrossed to wild type Oregon-R, *Df(1)446-20/+*, *Ppt1^A179T^/+*, *and Ppt1^S77F^/+*, all showed normal EVE expression (Tables 1 and 2). These results indicate that the EVE phenotypes are due to the loss of Ppt1.

**Table 1:** Percentage of hemisegments in *Ppt1*-deficient embryos with missing EVE+ RP2 neurons.

genotype	%	n
wild type	0	220
*Ppt1^Df(1)446-20^*	36.4	220
*Ppt1^Df(1)446-20^/+*	0	220
*Ppt1^A179T^*	16	198
*Ppt1^A179T^/+*	0	220
*Ppt1^S77F^*	17	198
*Ppt1^S77F^/+*	0	220
*Ppt1^S77F^/Ppt1^Df(1)446-20^*	12	220
*Ppt1^A179T^/Ppt1^Df(1)446-20^*	12	374

n, number of hemisegments at stage 14–16.

**Table 2:** Penetrance of EVE+ RP2 phenotype in *Ppt1*- embryos.

genotype	%	n
wild type	0	30
*Ppt1^Df(1)446-20^*	100	25
*Ppt1^Df(1)446-20^/+*	0	10
*Ppt1^A179T^*	50	18
*Ppt1^A179T^/+*	0	10
*Ppt1^S77F^*	32	28
*Ppt1^S77F^/+*	0	10
*Ppt1^S77F^/Ppt1^Df(1)446-20^*	27	37
*Ppt1^A179T^/Ppt1^Df(1)446-20^*	63	24

%, percentage of stage 15–16 embryos with 2 or more missing RP2s.

n, number of embryos scored.

**Figure 3:**
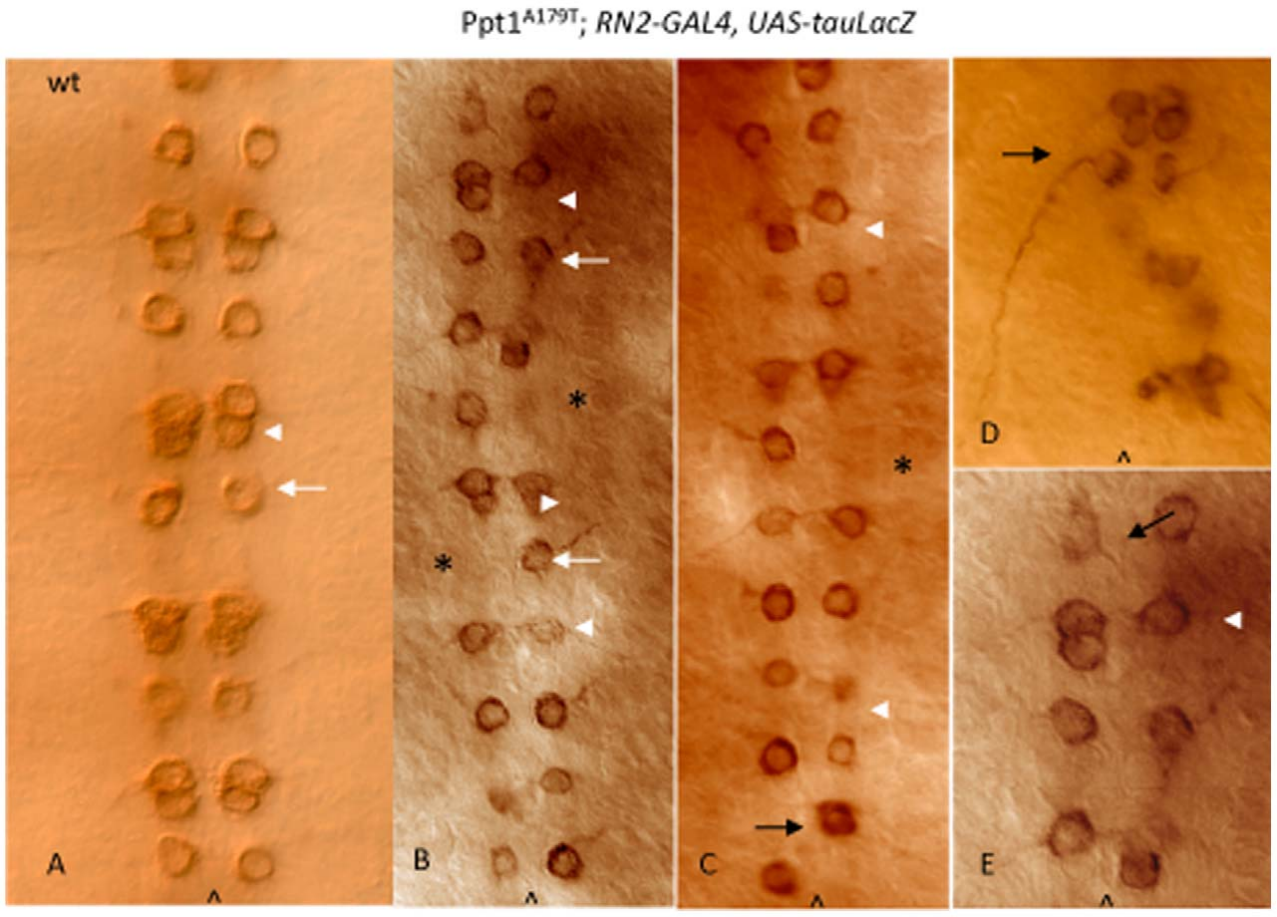
*Ppt1* mutants exhibit abnormal RP2 axon trajectories. S16 ventral nerve cord stained with anti-β-gal to detect RN2-*tau-lacZ* expressed in aCC, pCC and RP2 neurons. (A) Wild type RN2-*tau-lacZ* pattern showing RP2 neuron and its axon trajectory (white arrow); and aCC/pCC neurons (arrowhead). (B–D). *Ppt1^A179T^*; RN2-Gal4:UAS-*tauLacZ* embryos display a loss of RP2 motoneurons (asterisks) and aCC/pCC neurons (arrowheads) in many hemisegments; and disorganized cellular arrangements of the remaining LacZ-positive neurons. Many hemisegments have normal RP2 axon trajectory (white arrow) while others do not (black arrow). The RP2 in panel C shows an abnormal axon trajectory projecting posteriorly instead of anteriorly. Panel E is an enlarged subset of B. Anterior, up; caret, midline.

EVE is also essential for normal axonal guidance and innervation of the RP2 motoneuron [19] thus EVE can provide clues as to whether the observed alterations in cell fate lead to faulty axon pathfinding and innervation. To verify the mis-specification of neuronal cell fates in the EVE+ lineages as well as to assess the RP2 axonal projections, *Df(1)446-20* and *Ppt1^A179T^* flies were crossed to lines bearing either the *UAS-tau-LacZ* or *UAS-CD8-GFP* reporter (*RN2-Gal4:UAS-CD8-GFP or RN2-GAL4:UAS-tau-LacZ*) driven by the RP2/aCC/pCC-specific Gal4 driver (*RN2-Gal4*) to clearly mark the cell bodies and axons of aCC and pCC, and RP2 neurons [20]. Anti- β-gal staining and GFP visualization of embryos from these crosses confirmed EVE staining results. *Ppt1;RN2-Gal4:UAS-CD8-GFP* and *Ppt1; RN2-Gal4:UAS-tauLacZ* embryos display a loss of either *tau*LacZ or GFP-expressing RP2s and aCC/pCC neurons in many hemisegments; and disorganized cellular arrangements of the remaining LacZ-positive neurons. In many *Ppt1* mutants, the remaining RP2 motoneuron exhibits abnormal axon trajectory (Figure 3). Normally at late embryogenesis, the RP2 motoneuron extends its axon anteriorly toward the ipsilateral neighboring anterior segment to join the intersegmental nerve (ISN) to exit the CNS and eventually synapsing onto the dorsal muscle (Figure 3A). In many *Ppt1^A179T^; UAS-tauLacZ* embryos, the RP2 neurons extend its axons in the wrong direction-posteriorly to join the posterior ipsilateral ISN instead (Figure 3D) while other RP2 axons project contralaterally toward the midline, stalled, and never exit the CNS (Figure 3E). These embryos also display a loss of either aCC or pCC neurons in many hemisegments (Figure 3B–E). Collectively, these studies indicated that the loss of *Ppt1* caused abnormal neuronal cell fate specification and defective axon pathfinding.

### Embryonic glial development is normal in *Ppt1* mutants

Since neuronal and glial development are temporally and spatially linked and can be derived from a common neuronal precursor (e.g. NB1-1 produces both aCC/pCC neurons as well as the A and B glia located dorsally on the CNS [21] ), glial development was examined using the glial-specific marker REPO (mAb 8D12). The evolutionary conserved REPO protein is expressed in most glia in the embryonic CNS and PNS. During embryogenesis, REPO is essential for the migration and differentiation of glia, particularly the longitudinal glia of the CNS and the exit glia of the PNS. All *Ppt1* LOF mutants exhibit normal REPO-positive glia; thus *Ppt1* is not essential for the specification of embryonic glia (data not shown).

### *Ppt1* mutants exhibit variable embryonic CNS and PNS axonal defects

**Figure 4:**
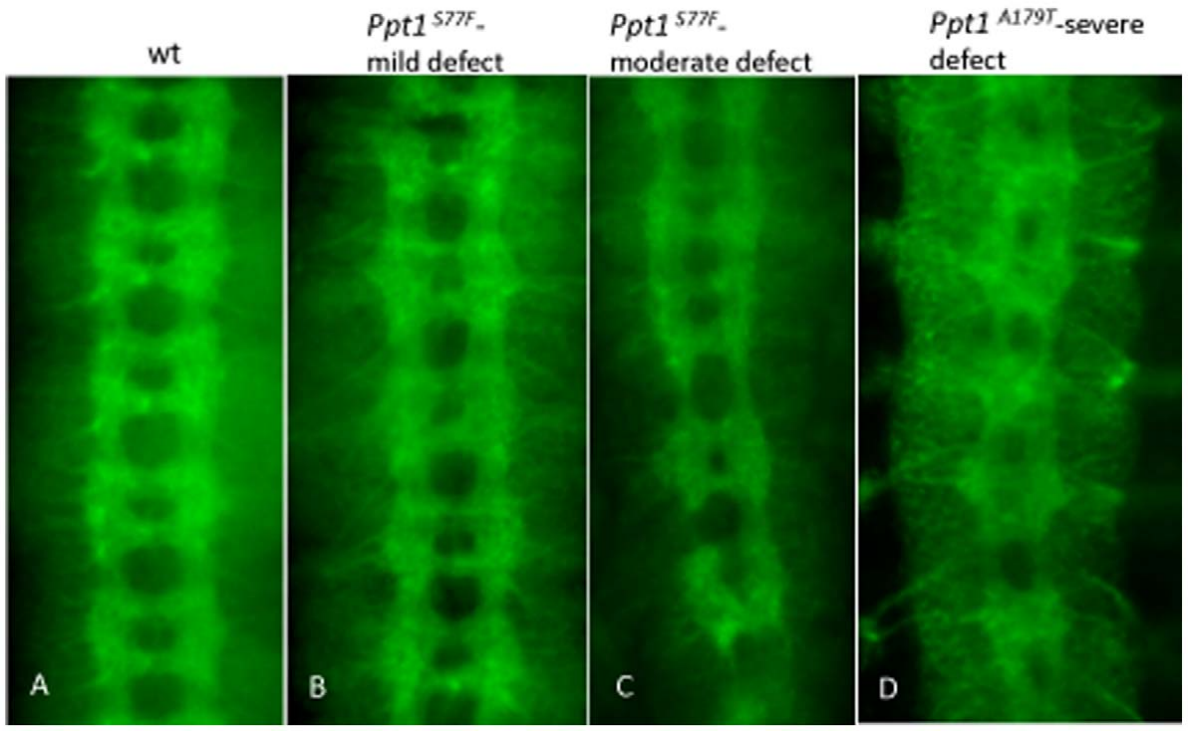
CNS scaffold abnormalities in *Ppt1* mutant embryos. In all panels, S16 embryos were stained with BP102. (A) Wild type embryo. (B) Axon defects range from mild phenotypes such as irregularly spaced axon tracts in commissures and wavy, thinning longitudinal connectives; to more severe phenotype such as fused commissures and disorganized CNS tracts (C and D). Anterior, up.

Overall axon guidance and fasciculation during neural development were examined using a number of axonal markers, BP102, FUTSCH (mAb22C10), and Fasciclin II (mAb1D4). In wild type, the axon tracts of the embryonic CNS consist of two longitudinal axon bundles running lengthwise along the ventral nerve cord intersected by segmentally reiterated pairs of anterior and posterior commissures that crosses the midline at every segment (Figure 4A). The CNS axon scaffold can be visualized using BP102 which recognizes a carbohydrate epitope present on all CNS axons [22]. 73% of *Df(1)446-20*, 35% of *Ppt1^A179T^*, and 24% of *Ppt1^S77F^* embryos show mild to severe defects (Figure 4; Table 3). Mild defects include irregularly spacing of the ventral nerve cord, some disorganization of the axon scaffold, abnormally thin or thick, and wavy longitudinal connectives. Moderate defects include narrowing of spaces between the commissures (Figure 4B), broader and/or more compressed anterior and posterior commissures, and interrupted, frayed longitudinal connectives (Figure 4C). Severe defects observed include severe disorganization of the longitudinal tracts at many segments, the complete loss of longitudinal connectives in other segments, and fused or missing commissures (Figure 4C & D).

**Table 3:** Percentage of *Ppt1*-deficient embryos displaying axonal defects.

Antibody staining	*Wild type*	*Df(1)446-20*	*Ppt1^A179T^*	*Ppt1^S77F^*
anti-BP102	0% (6)	73% (34)	35% (34)	12% (49)
anti-fasII (1D4)	0% (11)	58% (36)	50% (46)	n/d
Anti-FUTSCH (22C10)	0% (10)	70% (50)	60.5% (38)	n/d

Parentheses, the number of stage 16–17 embryos assayed.

n/d: not determined.

**Figure 5:**
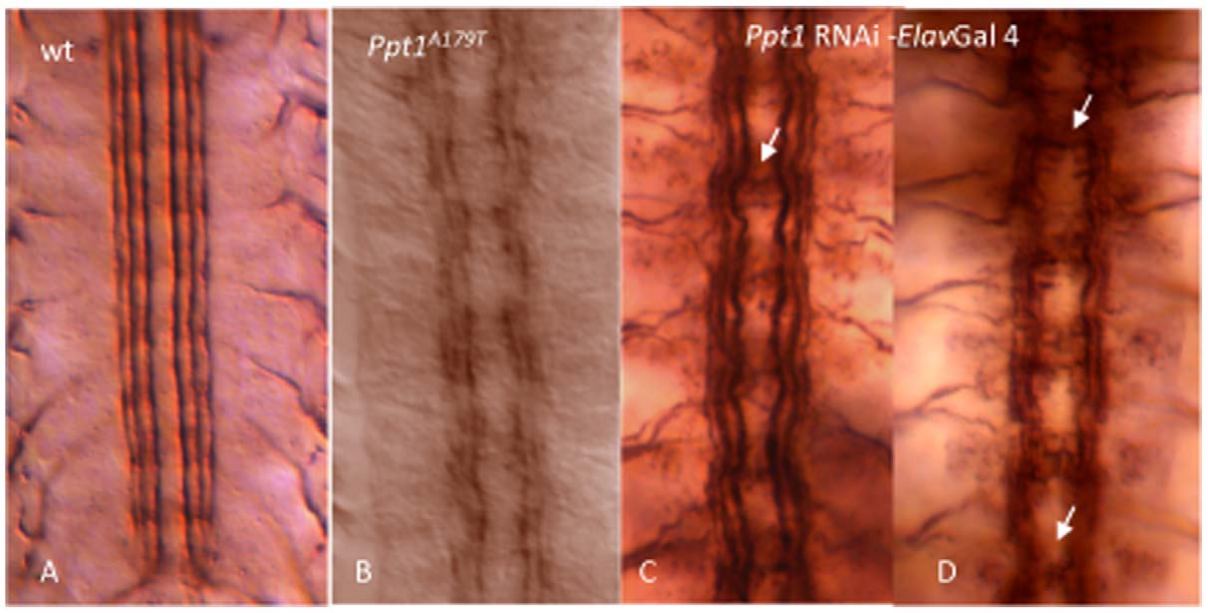
Mutant *Ppt1* and *Ppt1* RNAi knock-down in neurons result in defective axon tracts in the developing CNS. In all panels, S17 embryos were stained with 1D4 antibody. (A) FASII is expressed in three longitudinal connectives at this stage. (B) *Ppt1^A179T^* embryos exhibit discontinuous and frayed longitudinal connectives. (C, D) *Ppt1* RNAi expression under the control of *elav* gal4 driver results in a range of axon guidance phenotype including wavy bundles with some faulty axon crossing over the midline (arrow in C); and disorganized and/or broken connectives, and fused commissures (arrows in D). Anterior, up.

To evaluate the morphology of a more discrete subset of axonal longitudinal connectives, *Df(1)446-20*, *Ppt1^A179T^*, *and Ppt1^S77F^*, and trans-heterozygous *Ppt1* mutant embryos were stained with mAb1D4. At stage 17, mAb1D4 recognizes the neural adhesion protein Fasciclin II, which is expressed in three discrete axon longitudinal bundles on either side of the midline (Figure 5A; [23]). 50–58% of *Ppt1* mutant embryos display abnormal FasII-positive longitudinal connectives ranging from mild to severe (Table 3). Mild defects include loose and defasciculated connectives, wavy in appearance. More severe FasII defects include ectopic midline crossing of the longitudinal connectives, discontinuous connectives in all three FasII bundles, and frayed connectives due to defasciculation (Figure 5B).

We examine the specific requirement of *Ppt1* in neurons by driving the expression of *Ppt1*-RNAi in strains containing the ds RNA-GD3286 construct (obtained from the Vienna *Drosophila* RNAi Center [24]); and the dsRNA-JF01972 construct from the TriP-1 collection (Bloomington Drosophila Stock Center) using various neural-specific Gal4 drivers (Bloomington Drosophila Stock Center). The dsRNA-GD3286 construct recognizes 310 nucleotides within the first exon of the Ppt1 gene; the dsRNA-JF01972 construct recognizes 531 nucleotides-nearly all the first exon of the gene. Ppt1-RNAi embryos were examined for *Ppt1*-associated FasII defects. 85–100% *Ppt1*RNAi-*elav-*Gal4 embryos display defects ranging from mild to severe axon bundles (Figure 5C and D; Table 4). Similarly, 85% of *Ppt1*RNAi- U/CQ-Gal4 and 93% *Ppt1*RNAi-aCC/RP2-Gal4 embryos exhibit varying degrees of axon defects ranging from wavy but intact bundles; to frayed, discontinuous or missing bundles in many hemisegments. In contrast, embryos from the parental strains exhibit normal axon patterns (Table 5). These results collectively indicate that *Ppt1* is essential in the neurons for proper axon guidance and fasciculation.

**Table 4:** Quantitation of *Ppt1* RNAi embryos displaying FasII defects.

genotype	% defective
*Ppt1^5499^*RNAi	0.03 (30)
*Ppt1^25952^*TRiP RNAi	0 (30)
*elav*-gal4	0 (33)
UAS *tauLacZ CQ2-*gal4	0 (30)
UAS *tauLacZ RN2-*gal4	0 (35)
*Ppt1^5499^*RNAi×*elav*-gal4	100 (81)
*Ppt1^25952^*TRiP RNAi×*elav*-gal4	85.7 (42)
*Ppt1^5499^*RNAi×UAS *tauLacZ CQ2-*gal4	85 (67)
*Ppt1^5499^*RNAi×UAS *tauLacZ RN2-*gal4	93.4 (106)

Parentheses, the number of stage 16–17 embryos assayed.

*Ppt1* RNAi expression is driven by the following neural specific Gal4 drivers:

*elav-gal4-*drives expression in CNS and PNS neurons.

*UAS tau-LacZ* CQ2-gal 4 driver- drives tau-LacZ expression in U/CQ neurons.

*UAS tau-LacZ* RN2-gal 4 driver- drives *tau-LacZ* expression in aCC and RP2 neurons.

**Table 5:** Quantitation of *Ppt1* axon defects in the embryonic CNS and PNS.

phenotype	*Wild type*	*Ppt1^A179T^*	*Ppt1^446-20^*	*Ppt1^5499^*RNAi×*elav*-gal4	*Ppt1^25952^*TRiP RNAi×*elav*-gal4	*Ppt1^5499^*RNAi×UAS *tauLacZ CQ2*-gal4	*Ppt1^5499^*RNAi×UAS *tauLacZ RN2-*gal4
**PNS**							
Irregular spacing of lch5 neuron clusters	0	6	6	n/d	n/d	n/d	n/d
Missing lch5 neurons	0	4	6	n/d	n/d	n/d	n/d
Malformed lch5 neurons	0	13	13	n/d	n/d	n/d	n/d
**CNS**							
slightly irregular and/or wavy longitudinal tracts	0	31	21	40	9	25	7
Discontinuous, frayed longitudinal tracts	0	8	11	35	33	35	99
Irregular, missing or fused commissures	0	16	17	6	14	7	10
Total number of embryos scored	27	69	58	81	42	67	106

-number of embryos of each genotype displaying various fasII and 22C10 defects; many embryos exhibit combination of phenotypes.

n/d: not determined.

**Figure 6:**
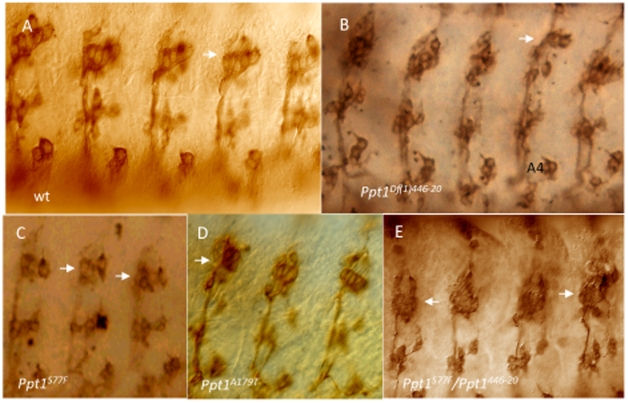
Chordotonal neurons (lch5) in the developing PNS are abnormal in many *Ppt1* LOF embryos. In all panels, lch5 neurons are indicated by the arrows. Side view of S17 embryos stained with Mab 22C10. (A) Wild type clusters of lch5, v, and v′ neurons are located in every abdominal segment. (B–E) lch5 neurons in mutant *Ppt1* embryos demonstrate a variety of defects: decreased number of neurons (B), fused and abnormally shaped (D, E), organization and positional defects, and thin axon bundles (black arrowhead) (B and C). dorsal, up.

The developing PNS of *Ppt1* mutants also show neuronal defects as assayed by 22C10, an antibody that recognizes the cytoskeletal FUTSCH protein found in the cytoplasm of a subset of CNS and PNS neurons and axons as well as their projections [25]. Normally, FUTSCH is expressed in the cell bodies as well as the axons and dendrites of 44 PNS neurons per abdominal segment [26]. 22C10-positive PNS neurons include the dorsal cluster (d), the ventral and ventral' cluster (v and v′), and the lateral cluster which consists of five chordotonal organs (lch5). Wild type and *Ppt1* LOF embryos showed no detectable difference in the number of neurons or axon projections in the l, v, and v′ neuron clusters. However, the lch5 neurons showed a variety of defects including decreased number of sensory neurons, fused and abnormally shaped neurons, and disorganized clusters with aberrant dendritic projections (Figure 6; Table 5). Occasionally, fasciculation defects with abnormal axonal connections between **v′** clusters and ISN are observed (data not shown). These findings indicate that lch5 neuron clusters are preferentially more sensitive than l, v, and v′ clusters to the loss of *Ppt1*.

## Discussion

RNA and antibody expression results reveal the low abundance nature of Ppt1 RNA and protein confirming what was previous thought- Ppt1 is an essential housekeeping gene. Because of its low abundance, we are unable to ascertain the subcellular localization of Ppt1 and thus could not determine whether fly Ppt1 is lysosomal, synaptic, or both. To address this question, Ppt1 would need to be over-expressed by at least 17-folds to be at a marginally detectable level using immunofluorescence. This is not ideal since Ppt1 over-expression leads to apoptosis. Our studies clearly indicate that Ppt1 protein is expressed ubiquitously at a very low level such that only a minute amount is required for function. Whether this minute amount of protein resides predominantly in the lysosomes and/or synapses for the turnover of palmitoylated developmental proteins, it is necessary and its absence would most likely have similar consequences.

Our results indicate that *Ppt1* is essential at a time when neural precursors are being determined, one of the earliest stages of embryonic neural development. Small discreet defects were detected early shortly after gastrulation with the alteration in CNS neural precursor cell fates in a number of identified neural precursors. Specifically, the phenotype is limited to a discreet subset of EVE+ neural lineages (e.g. the neuroblast lineage giving rise to RP2s, aCC and pCC neurons); while other EVE+ cells such as U's and EL interneurons are normal. In the affected lineages, the loss of *Ppt1* has small consequences at the early cell determination stage such that only a subset of all EVE+ RP2s, aCC/pCC neurons are affected. This indicates that even at this very early stage of neurogenesis, Ppt1-mediated protein turnover is essential. Neural defects increase as development proceeds such that by mid-to–late embryogenesis, malformed neurons and abnormal longitudinal axon tracts are visible in nearly all *Ppt1-*deficient embryos. Specifically, the development of the RP2, aCC and pCC neurons requires *Ppt1*; most likely through regulating EVE expression. Without *Ppt1*, many RP2, aCC/pCC neurons are abnormally positioned or missing; while some exhibit aberrant axon projections. Since EVE regulates proper axon projections in the developing CNS, particularly dorsally projecting aCC and RP2 motoneurons (pCC is an interneuron), this phenotype is wholly unexpected. These pathfinding and fasciculation defects reflect what we suspect: Ppt1-mediated turnover of palmitoylated developmental proteins is necessary throughout development such that its loss is cumulative and progressive. This is supported by previous studies showing that young adult brains exhibit age-dependent abnormal autofluorescence deposits due to the absence of *Ppt1* [12]. Neurons, in particular, exhibit a high rate of palmitoylation-depalmitoylation dynamics due to its high level of signal transduction and protein trafficking through neurotransmitter release. Since there are numerous endosomal/lysosomal pathways, the loss of one protein would not necessarily result in massive disturbances but may limit the number of protein turnovers and recycling which may cause a “domino” effect by directly or indirectly disrupting downstream pathways during neural development.

Downstream targets of *Ppt1* during neurogenesis are largely unknown. A previous study indicates that FasII, a neural adhesion protein, may be a candidate [14]. *Ppt1* over-expression screens have isolated FasII as a potential *Ppt1*-interacting protein. Our results showed that *Ppt1*-deficient embryos display FasII-specific axon fasciculation defects. FasII, a homolog of the vertebrate NCAM, is a palmitoylated protein and serves as a second anchor for proteins involved in N-CAM mediated signaling at the membrane [27]. Fasll has also been shown to interact with lipid rafts to mediate FGF signaling to promote neurite outgrowth [28]. Perhaps Fasll plays a similar role during fly neurogenesis where it is involved in lipid raft-mediated signaling. Since *Ppt1* has been suggested to also facilitate cell signaling via its localization to lipid rafts [29], FasII may require *Ppt1*-mediated palmitoylation turnover at the membrane to regulate the integration of signaling required to insure proper axon outgrowth and fasciculation.

The loss of *Ppt1* has varied consequences from the loss or gain of neurons, axon fasciculation defects, to axon guidance in a subset of motor neurons (e.g. RP2, aCC), sensory neurons, and interneurons (e.g. pCC but not the EL interneurons). This observation is analogous to what is observed in INCL humans and animal models. INCL patients undergo progressive degeneration in selective areas of the brain while leaving others intact. PPT1-deficient adult mice exhibit the loss of GABAergic interneurons in discrete regions of the brain [10] and thalamic neurons [30] months prior to being symptomatic. It is presently unclear whether PPT1-deficiencies affect mice earlier, during embryonic neural development.

The low penetrance of *Ppt1* phenotype indicates that the loss of *Ppt1* is generally well tolerated perhaps due to the presence of compensatory mechanisms. A closely related sister gene, PPT2, exists in mammals [31] and in *Drosophila*. PPT2 knock-out mice display many of the same INCL phenotype and pathology including spasticity and autofluorescence accumulation in the brain [32]. We are currently investigating whether the PPT2 fly homolog, *Ppt2*, also have an embryonic neural phenotype and whether *Ppt1/Ppt2* double mutants have a more severe phenotype than the single mutation.

## Materials and Methods

### *Drosophila* Strains

*Ppt1* LOF mutant alleles *Ppt1^A179T^*, *Ppt1^S77F^*, and the *Df(1)446-20* deficiency line were provided by Dr. Robert Glaser (Wadsworth Center, NYS Department of Health, Albany, NY). Oregon-R, w^*^; P{RN2-GAL4}P, P{UAS-tau-lacZ.B}2 and w^*^; P{RN2-GAL4}P, P{UAS-mCD8::GFP.L}LL5 , *Elav*-Gal4 driver, U/CQ-Gal4 driver, aCC/RP2-Gal4 driver, and *Ppt1*-RNAi TRiP line, and Oregon-R were obtained from Bloomington Drosophila Stock Center. *Ppt1*- UAS RNAi flies (w1118; P{GD3286} [14]) was obtained from Vienna *Drosophila* RNAi Center (Vienna, Austria). All strains were maintained on standard *Drosophila* cornmeal-yeast medium at 25°C.

To detect autofluorescence deposits, brains from 14-day old Oregon-R and *Ppt1^S77F^* were extracted and immediately examined on a Leica SP2 confocal microscope for comparison.

### Immunocytochemistry and Immunofluorescence

Embryos were fixed and stained according to previously described methods [33], [34], and using the following primary antibodies obtained from University of Iowa Developmental Studies Hybridoma Bank: mAb 22C10 (1∶50), mAb BP102 (1∶50), mAb 2B8 (1∶30), and mAb 1D4 (1∶5). β-gal monoclonal antibody at 1∶1000 obtained from Jackson ImmunoResearch, Inc. Immunocytochemical detection was performed using HRP-conjugated goat anti-mouse secondary antibody (1∶300; Jackson ImmunoResearch, Inc.). Immunofluorescence was performed using Alexa-488 and Alexa-594 conjugated goat anti-mouse secondary antibodies (1∶300; Molecular Probes/ Invitrogen, Inc.). For each genotypic staining of EVE, 100–200 thoracic and abdominal hemisegments from at least 20 appropriately staged embryos were counted. Digital images of stained embryos were captured using either the Advanced Color SPOT system or the Retiga EXi Fast 1394 mounted onto a Nikon E600 compound microscope.

## Supporting Information

Figure S1 Whole mount *in situ* RNA hybridization reveals that *Ppt1* transcript is ubiquituously expressed at low levels during (A) embryogenesis and (B) 3rd instar imaginal discs. 0–16 hours wild type embryos and 3rd instar imaginal discs were collected, fixed and hybridized with digoxygenin-labeled anti-sense *Ppt1* RNA probes. Anti-sense RNA probes were generated from a CG12108 cDNA clone GM21019 (Research Genetics). Embryo *in situ* hybridization was performed using standard protocols.
(0.82 MB PDF)

Figure S2 Western blot using affinity-purified Ppt1 polyclonal antibody (generated against the C-terminus) of S2 cell lysate, wild type W1118 fly head extract (arrows), and over-expressed Gal4-UAS-Ppt1 head extract untreated (A) and treated with pGNAse (B) to remove N-glycosylation. Results indicate that although no band is detected in the S2 lysate and W1118 lanes (arrows), Ppt1 antibody is specific to Ppt1 protein and can be detected when Ppt1 is over-expressed (A and B). Two rabbit and two chicken polyclonal antibodies were generated using the following peptides located in the internal region of the *Ppt1* gene: VAERCPNPPMRNLIT, ATYWHDPIMENKYR, and IVQPKESQWFQYYTT ; and VYQNLGLDKMHRQGQ located at the C-terminal region (GeneMed Synthesis, Inc.; Aves Laboratories, Inc.). Affinity purifications were performed using the Pierce Biotechnology SulfoLink (#20405) and AminoLink kit (#44890) according manufacturer's standard protocols. Western blot was performed using the ECL Western Blotting Analysis System (Pierce Biotechnology). Deglycosylation experiments were performed using lysates prepared from S2 Schneider cells, and adult fly heads from W1118 and UAS:Ppt1 2.1/GMR:GAL4 flies. Samples were treated with PGNase F according to manufacturer's instruction (glycerol free kit #P0705S; New England Biolabs, Inc.) prior to Western Blotting.
(0.29 MB PDF)
