# Supplementary figures and images for: The Batten Disease Palmitoyl Protein Thioesterase 1 Gene Regulates Neural Specification and Axon Connectivity during Drosophila Embryonic Development
Source: PLoS One. 2010 Dec 22;5(12):e14402. doi: 10.1371/journal.pone.0014402 (PMC3008717; doi:10.1371/journal.pone.0014402)

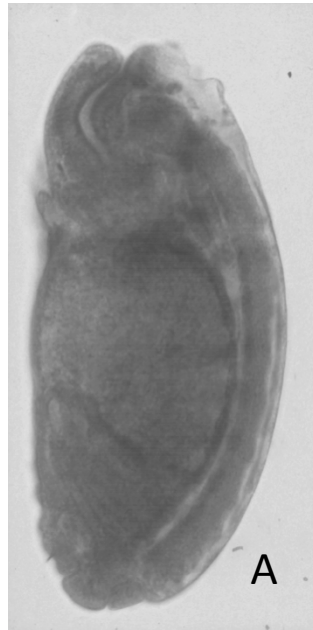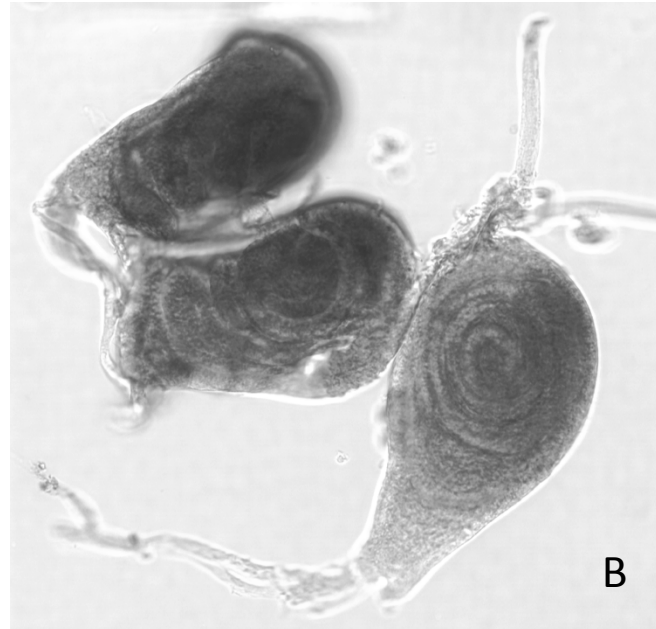

Supplement: Figure S1 — Whole mount in situ RNA hybridization reveals that Ppt1 transcript is ubiquituously expressed at low levels during (A) embryogenesis and (B) 3rd instar imaginal discs. 0–16 hours wild type embryos and 3rd instar imaginal discs were collected, fixed and hybridized with digoxygenin-labeled anti-sense Ppt1 RNA probes. Anti-sense RNA probes were generated from a CG12108 cDNA clone GM21019 (Research Genetics). Embryo in situ hybridization was performed using standard protocols. (0.82 MB PDF) [file pone.0014402.s001.pdf]

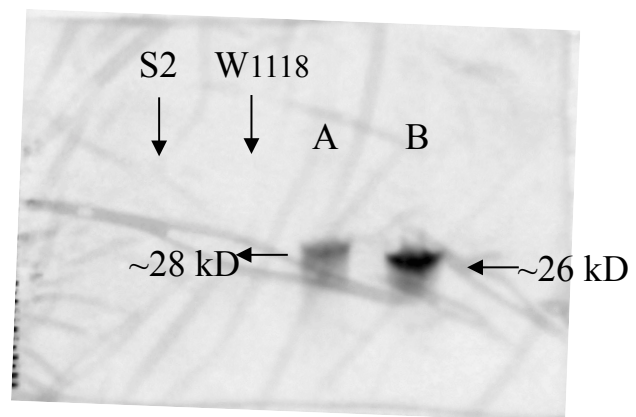

Supplement: Figure S2 — Western blot using affinity-purified Ppt1 polyclonal antibody (generated against the C-terminus) of S2 cell lysate, wild type W1118 fly head extract (arrows), and over-expressed Gal4-UAS-Ppt1 head extract untreated (A) and treated with pGNAse (B) to remove N-glycosylation. Results indicate that although no band is detected in the S2 lysate and W1118 lanes (arrows), Ppt1 antibody is specific to Ppt1 protein and can be detected when Ppt1 is over-expressed (A and B). Two rabbit and two chicken polyclonal antibodies were generated using the following peptides located in the internal region of the Ppt1 gene: VAERCPNPPMRNLIT, ATYWHDPIMENKYR, and IVQPKESQWFQYYTT ; and VYQNLGLDKMHRQGQ located at the C-terminal region (GeneMed Synthesis, Inc.; Aves Laboratories, Inc.). Affinity purifications were performed using the Pierce Biotechnology SulfoLink (#20405) and AminoLink kit (#44890) according manufacturer's standard protocols. Western blot was performed using the ECL Western Blotting Analysis System (Pierce Biotechnology). Deglycosylation experiments were performed using lysates prepared from S2 Schneider cells, and adult fly heads from W1118 and UAS:Ppt1 2.1/GMR:GAL4 flies. Samples were treated with PGNase F according to manufacturer's instruction (glycerol free kit #P0705S; New England Biolabs, Inc.) prior to Western Blotting. (0.29 MB PDF) [file pone.0014402.s002.pdf]
